# Alterations of the Gut Microbiota in Patients with Diabetic Nephropathy

**DOI:** 10.1128/spectrum.00324-22

**Published:** 2022-07-14

**Authors:** Lili Zhang, Zhisheng Wang, Xiaona Zhang, Lu Zhao, Jinjin Chu, Haibo Li, Wenchang Sun, Chunjuan Yang, Hui Wang, Wenqing Dai, Shushan Yan, Xiaohua Chen, Donghua Xu

**Affiliations:** a Department of Central Laboratory, The First Affiliated Hospital of Weifang Medical University, Weifang, China; b Department of Neurosurgery, Sunshine Union Hospital, Weifang, China; c Department of Endocrinology, The First Affiliated Hospital of Weifang Medical University, Weifang, China; d Department of Biostatistics, School of Public Health, Cheeloo College of Medicine, Shandong University, Jinan, China; e Department of Rheumatology, The First Affiliated Hospital of Weifang Medical University, Weifang, China; f Department of General Surgery, The Affiliated Hospital of Weifang Medical University, Weifang, China; g Department of Nuclear Medicine, The First Affiliated Hospital of Weifang Medical University, Weifang, China; Lerner Research Institute

**Keywords:** gut microbiota, diabetic nephropathy, metagenomics, composition, function

## Abstract

Diabetic nephropathy (DN) is the primary cause of end-stage renal disease. Accumulating studies have implied a critical role for the gut microbiota in diabetes mellitus (DM) and DN. However, the precise roles and regulatory mechanisms of the gut microbiota in the pathogenesis of DN remain largely unclear. In this study, metagenomics sequencing was performed using fecal samples from healthy controls (CON) and type 2 diabetes mellitus (T2DM) patients with or without DN. Fresh fecal samples from 15 T2DM patients without DN, 15 DN patients, and 15 age-, gender-, and body mass index (BMI)-matched healthy controls were collected. The compositions and potential functions of the gut microbiota were estimated. Although no difference of gut microbiota α and β diversity was observed between the CON, T2DM, and DN groups, the relative abundances of butyrate-producing bacteria (*Clostridium*, *Eubacterium*, and Roseburia intestinalis) and potential probiotics (*Lachnospira* and *Intestinibacter*) were significantly reduced in T2DM and DN patients. Besides, Bacteroides stercoris was significantly enriched in fecal samples from patients with DN. Moreover, *Clostridium* sp. 26_22 was negatively associated with serum creatinine (*P* < 0.05). DN patients could be accurately distinguished from CON by *Clostridium* sp. CAG_768 (area under the curve [AUC] = 0.941), Bacteroides propionicifaciens (AUC = 0.905), and *Clostridium* sp. CAG_715 (AUC = 0.908). DN patients could be accurately distinguished from T2DM patients by *Pseudomonadales*, Fusobacterium varium, and *Prevotella* sp. MSX73 (AUC = 0.889). Regarding the potential bacterial functions of the gut microbiota, the citrate cycle, base excision repair, histidine metabolism, lipoic acid metabolism, and bile acid biosynthesis were enriched in DN patients, while selenium metabolism and branched-chain amino acid biosynthesis were decreased in DN patients.

**IMPORTANCE** Gut microbiota imbalance is found in fecal samples from DN patients, in which Roseburia intestinalis is significantly decreased, while Bacteroides stercoris is increased. There is a significant correlation between gut microbiota imbalance and clinical indexes related to lipid metabolism, glucose metabolism, and renal function. The gut microbiota may be predictive factors for the development and progression of DN, although further studies are warranted to illustrate their regulatory mechanisms.

## INTRODUCTION

Diabetic nephropathy (DN) is a chronic kidney disease (CKD), which is one of the most common complications of diabetic microangiopathy and the primary cause of end-stage renal disease (ESRD) (1). The incidence of diabetes mellitus (DM) and DN has been increasing in the last decade. According to data from the International Diabetes Federation, the number of diabetics is up to 425 million worldwide nowadays and is predicted to rise to 700.2 million by 2045 (2). About 30% to 40% of DM patients can develop into DN, and one-third of DN patients further develop ESRD (3, 4). The mortality of DN patients is 30 times higher than that of DM patients without kidney disease (5). Accordingly, DN seriously threatens people’s life and health.

The pathogenesis of DN is complicated and remains largely unknown. It has been well established that abnormal glucose and lipid metabolism caused by hyperglycemia (6), hemodynamic changes (7), mitochondrial dysfunction (8), an activated renin-angiotensin-aldosterone signaling pathway (9), immune disorders (10), oxidative stress (11), and genetic susceptibility (12) contribute to renal dysfunction and thus DN. Increasing evidence has suggested that the imbalance of the gut microbiota participates in DN pathogenesis (13). The gut microbiota is composed of approximately 10^13^ to ~10^14^ bacteria and is well known as the “second genome” of human body. The gut microbiota affects the intestinal barrier, renal metabolism, inflammation, and immune microenvironment balance (14). Gut microbiota imbalance is closely related to obesity, DM, and other metabolic diseases (15). During the past few years, the role of the gut microbiota in DN has been drawing more and more attention. Imbalanced gut microbiota has been demonstrated in fecal samples from DN patients, including increased abundances of *Proteobacteria*, *Verrucomicrobia*, and *Fusobacteria* (16). Most interestingly, the abundance of particular organisms in the gut microbiota in DN patients is significantly different from that in DM patients without DN, such as Escherichia-*Shigella* and *Prevotella* (17). Moreover, increased metabolic toxins in DN patients due to impaired renal function and decreased excretion can lead to the imbalance of the gut microbiota, which further aggravates the progression of DN (18). In addition, a number of studies have found that some metabolites from the gut microbiota play important roles in DN, such as lipopolysaccharide (LPS) (19), short-chain fatty acids (SCFAs) (20), bile acids (BAs) (21), and trimethylamine-*N*-oxide (TMAO) (22). However, the underlying molecular mechanism of the gut microbiota involved in the pathogenesis of DN is still unclear.

In the present study, we aimed to investigate the characteristics of the gut microbiota and the potential microbiome functions in DN through metagenome sequencing analysis. The main driving bacteria in DN are explored, including their correlation with the clinical indexes of glucose metabolism and renal function.

## RESULTS

### Clinical baseline information of participants.

After excluding the unqualified samples, 14 healthy controls (CON), 12 patients with type 2 diabetes mellitus (T2DM), and 12 DN patients were included into this study. Fasting blood glucose (FBG), glycosylated hemoglobin (HbA1c), body mass index (BMI), total cholesterol (TC), total triglyceride (TG), high-density lipoprotein cholesterol (HDL-C), low-density lipoprotein cholesterol (LDL-C), the urinary microalbumin/urinary creatinine ratio (ACR), urinary microalbumin (ALB), urinary creatinine (UCR), uric acid (UA), urea, serum creatinine (SCR), and the urea/creatinine ratio (BUN/CR) were presented as the crucial indexes for the diagnosis of T2DM or DN (Table 1). There was no statistical significance between the three groups regarding age, gender, BMI, SCR, BUN/CR, and blood lipid levels. Levels of FBG, HbA1c, ACR, ALB, UA, and urea were increased obviously, while the level of UCR was significantly decreased in the DN group compared with the CON group. The levels of ACR and ALB of DN patients were significantly higher than those of T2DM patients.

**TABLE 1:** Baseline clinical indexes of participants

Characteristic	Value for indicated group		
	CON (*n* = 14)	T2DM (*n* = 12)	DN (*n* = 12)
Age (yrs)	58.86 ± 7.36	57.08 ± 8.59	61.67 ± 8.75
Female gender, no. (%)	7 (50)	5 (41.67)	6 (50)
FBG (mmol L^−1^)	4.98 ± 0.51	8.21 ± 2.91^*a*^	8.3 ± 1.62^*a*^
HbA1C (%)	4.89 ± 0.48	9.17 ± 2.08^*a*^	8.79 ± 2.00^*a*^
TC (mmol L^−1^)	4.22 ± 1.17	4.67 ± 1.39	4,75 ± 1.53
TG (mmol L^−1^)	1.15 ± 0.32	2.12 ± 2.48	2.05 ± 2.00
HDL-C (mmol L^−1^)	1.27 ± 0.20	1.19 ± 0.39	1.25 ± 0.37
LDL-C (mmol L^−1^)	3.32 ± 0.72	2.62 ± 1.05	2.64 ± 1.08
BMI (kg m^2 −1^)	24.68 ± 2.48	23.46 ± 7.08	27.56 ± 3.39
ACR	9.76 ± 2.76	18.82 ± 20.40	228.85 ± 280.99^*a*^^*b*^
ALB (mg L^−1^)	15.21 ± 7.01	19.39 ± 20.60	84.91 ± 86.21^*a*^^*b*^
UCR (mg dL^−1^)	151.48 ± 40.33	118.27 ± 64.92	72.58 ± 62.48^*a*^
UA (μmol L^−1^)	239.50 ± 46.48	313.41 ± 105.41^*a*^	348.44 ± 71.05^*a*^
Urea (mmol L^−1^)	5.29 ± 0.62	6.09 ± 0.97	6.72 ± 1.66^*a*^
SCR (μmol L^−1^)	52.96 ± 12.38	58.07 ± 7.23	66.58 ± 29.97
BUN/CR	0.10 ± 0.03	0.11 ± 0.02	0.11 ± 0.03

a *P* < 0.05, compared with the CON group.

b *P *< 0.05, compared with the T2DM patients.

### Composition of gut microbiota between the CON, T2DM, and DN groups.

**FIG 1:**
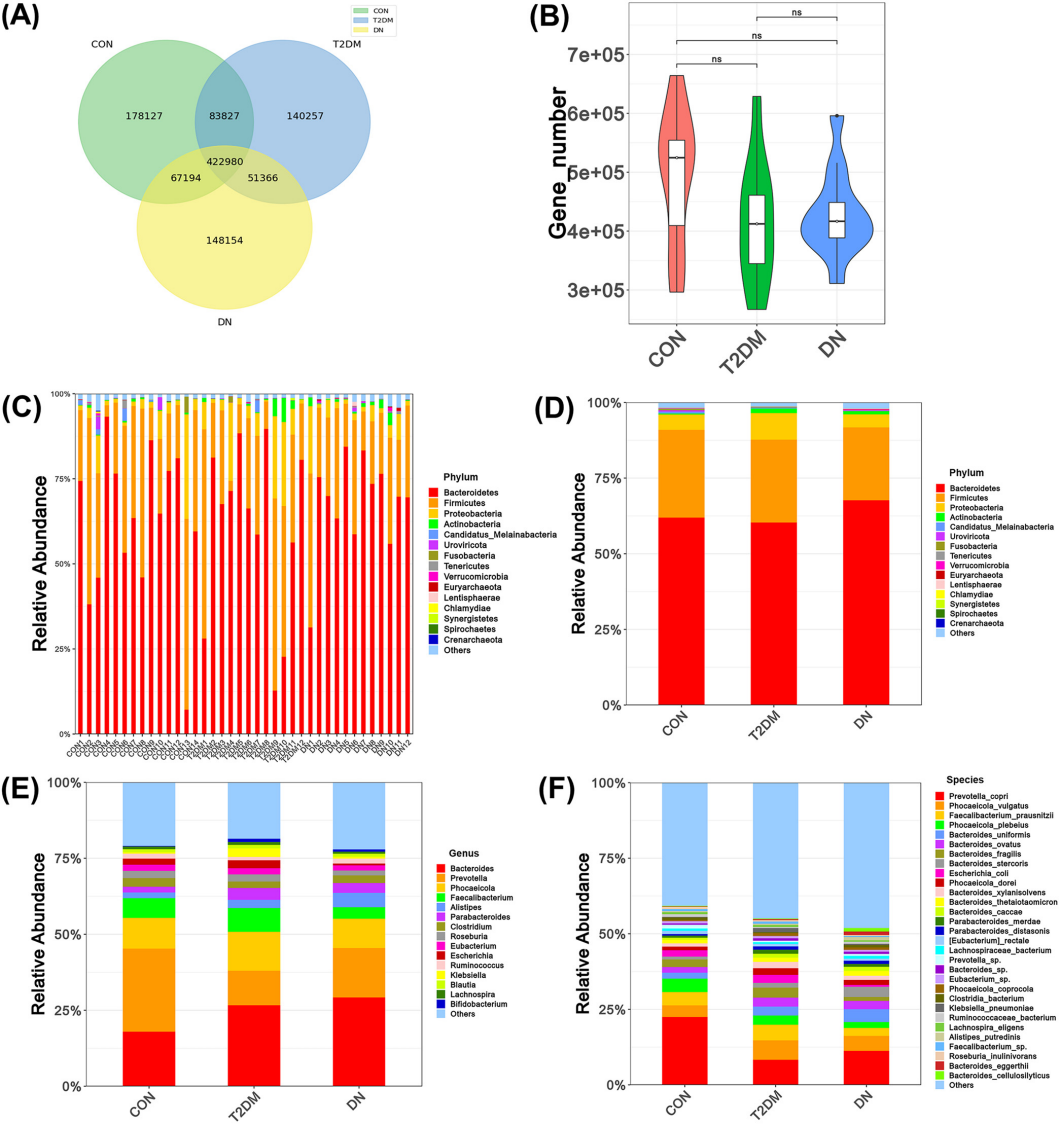
Gut microbiota compositions of the CON, T2DM, and DN groups. (A) Venn diagram of gene numbers in the CON, T2DM, and DN groups. (B) Wilcoxon rank sum test of gene numbers in CON, T2DM, and DN patients. (C) Gut microbiota composition at the phylum level of each sample. (D) Gut microbiota composition at the phylum level. (E) Gut microbiota composition at the genus level. (F) Gut microbiota composition at the species level.

After data filtering and assembly, the effective data size of each sample was distributed in the range of 5.56G to 8.89G. The *N*_50_ statistics of contigs were in the range of 1,679 to 10,288 bp. A total of 1,091,908 gene catalogues were constructed after deredundancy. Venn diagram analysis showed that the three groups consisted of 422,980 genes, with 178,127 unique genes in the CON group, 140,257 unique genes in the T2DM group, and 148,154 unique genes in the DN group (Fig. 1A). There were no statistical differences in the numbers of genes among the three groups (Fig. 1B). After the sequence alignment and annotation, the abundance of species at each taxonomic level (phylum, class, order, family, genus, and species) was ranked. The relative abundance of the top 15 bacteria at the phylum level in each sample was displayed. There were significant individual differences among the gut microbiotas within each group (Fig. 1C). In addition, we analyzed the relative abundance of the top 15 bacteria of the gut microbiota in the CON, T2DM, and DN groups at three taxonomic levels: phylum, genus, and species. At the phylum level (Fig. 1D), 95% of the bacteria were mainly composed of *Bacteroidetes*, *Firmicutes*, *Proteobacteria*, and *Actinobacteria*. *Bacteroidetes* was the dominant phylum, accounting for more than 60% of each group (CON, 61.94 ± 0.23; T2DM, 60.30 ± 0.26; and DN, 67.64 ± 0.14). There were no statistical differences regarding the relative abundances of the top 15 species at the phylum level. The ratio of *Bacteroidetes*/*Firmicutes* (B/F) was commonly used to assess changes in phylum levels of the gut microbiota. In this study, the B/F ratios of the CON, T2DM, and DN groups were 2.14, 2.2, and 2.8, respectively, which suggested a gradual increase of B/F ratio in T2DM and DN groups but without statistically significance. At the genus level (Fig. 1E), 60% of the species were composed of *Bacteroides*, *Prevotella*, *Phocaeicola*, *Faecalibacterium*, and *Alistipes*. In the CON group, the proportion of *Prevotella* (27.31%) was the highest. However, *Bacteroides* was predominant in the T2DM and DN groups, accounting for 26.67% and 29.22%, respectively. Moreover, the relative abundance of *Lachnospira* showed statistical significance among the three groups (*P < *0.05) (see Table S2 in the supplemental material). At the species level, the proportions of the top 30 species in the CON, T2DM, and DN groups were decreased 58.35%, 54.48%, and 51.01%, respectively (Fig. 1F). The relative abundances of Bacteroides stercoris, Bacteroides fragilis, and Bacteroides eggerthii in the three groups was statistically significant (*P < *0.05) (Table S3). The most abundant species in each group was Prevotella copri. In addition, the proportions of Prevotella copri in the CON, T2DM, and DN groups were 22.13%, 8.24%, and 11.08%, respectively.

### Gut microbiota diversity between the CON, T2DM, and DN groups.

**FIG 2:**
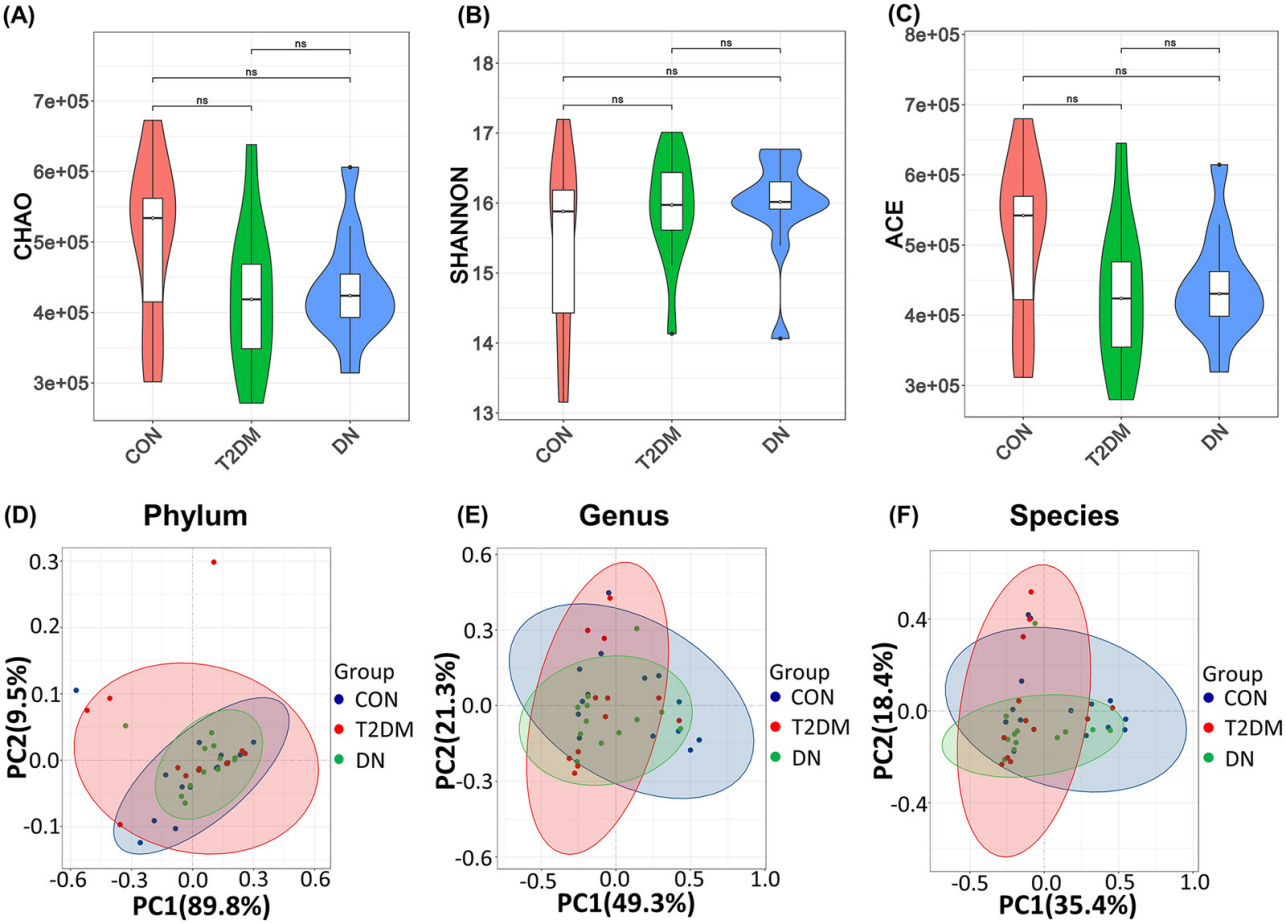
Diversity of the gut microbiota in the CON, T2DM, and DN groups. (A to C) Chao, Shannon, and ACE indexes of gut microbiota α diversity in the CON, T2DM, and DN groups. (D to F) PCoA analysis of gut microbiota β diversity in the CON, T2DM, and DN groups at the phylum, genus, and species levels.

We used Chao, Shannon, and ACE indexes to assess α diversity (richness and diversity of the gut microbiota) of the bacterial community (Fig. 2A to C). There were no obvious changes in α diversity of the bacterial community among the three groups. The β diversity of the gut microbiota was evaluated by principal-coordinate analysis (PCoA) based on the Bray-Curtis distance matrix at the levels of phylum (Fig. 2D), genus (Fig. 2E), and species (Fig. 2F). Analysis of similarity (ANOSIM) of the β diversity had demonstrated that there were no significant differences between the three groups in the fecal microbial communities (phylum, *r* = −0.021 and *P *= 0.695; genus, *r* = 0.013 and *P *= 0.306; and species, *r* = 0.037 and *P *= 0.161). These results showed that the disease status might not influence the community richness and diversity of the gut microbiota.

### Differential species of the gut microbiota in the CON, T2DM, and DN groups.

**FIG 3:**
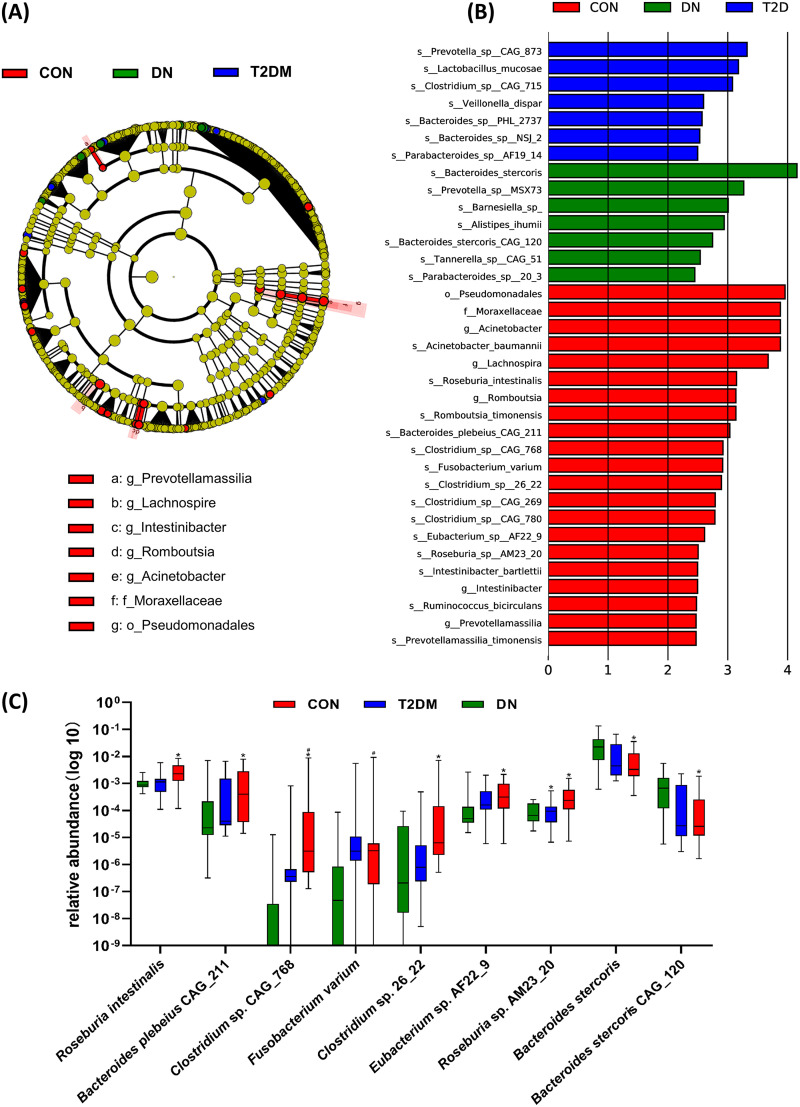
Differential species of gut microbiota between the CON, T2DM, and DN groups. (A) Annotated branch diagram of the different species of the gut microbiota in CON, T2DM, and DN patients. (B) LEfSe analysis showing the most differentially abundant taxa between the CON, T2DM, and DN groups. Red, blue, and green bars represented species with relatively higher abundance in CON, T2DM, and DN patients, respectively. Only species with an LDA  of >2 are shown. (C) Increased or decreased strains in the CON, T2DM, and DN groups. *, *P < *0.05, compared with CON group; *#*, *P < *0.05, compared with T2DM group.

A linear discriminant analysis effect size (LEfSe) analysis was used to screen the differential biomarkers of the gut microbiota in each group to explore the specific bacteria associated with DN. The taxonomic cladogram showed the dominant species of gut microbiota in the three groups from phylum level to genus level (Fig. 3A). We marked 35 distinguishing taxa with differential abundances among the groups by linear discriminant analysis (LDA) scores above 2.0. (Fig. 3B). In the CON group, some bacteria were significantly higher than those in the T2DM and DN groups, including the order *Pseudomonadales*, the family *Moraxellaceae*, the genera Acinetobacter, *Lachnospira*, *Romboutsia*, *Intestinibacter*, and *Prevotellamassilia*, and the species Acinetobacter baumannii, Roseburia intestinalis, *Romboutsia timonensis*, Bacteroides plebeius CAG_211, *Clostridium* sp. CAG_768, Fusobacterium varium, *Clostridium* sp. 26_22, *Clostridium* sp. CAG_269, *Clostridium* sp. CAG_780, *Eubacterium* sp. AF22_9, *Roseburia* sp. AM23_20, Intestinibacter bartlettii, Ruminococcus bicirculans, and *Prevotellamassilia timonensis*. Seven strains (*Prevotella* sp. CAG_873, Lactobacillus mucosae, *Clostridium* sp. CAG_715, Veillonella dispar, *Bacteroides* sp. PHL_2737, *Bacteroides* sp. NSJ_2, and *Parabacteroides* sp. AF19_14) were significantly enriched in the gut microbiota of T2DM patients. We had also found that the relative abundances of 7 strains in DN patients were significantly higher than those in other groups, including Bacteroides stercoris, *Prevotella* sp. MSX73, *Barnesiella*, Alistipes ihumii, Bacteroides stercoris CAG_120, *Tannerella* sp. CAG_51, and *Parabacteroides* sp. 20_3. In particular, the LDA value of *B. stercoris* exceeded 4. Furthermore, seven strains (Roseburia intestinalis, Bacteroides plebeius CAG_211, *Clostridium* sp. CAG_768, Fusobacterium varium, *Clostridium* sp. 26_22, *Eubacterium* sp. AF22_9, and *Roseburia* sp. AM23_20) showed a decrease in the T2DM and DN groups. Two strains (*B. stercoris* and *B. stercoris* CAG_120) were increased from the CON group and from the T2DM group to the DN group, which might be related to the progression of DN (Fig. 3C).

### Correlation between differential species and clinical indexes.

**FIG 4:**
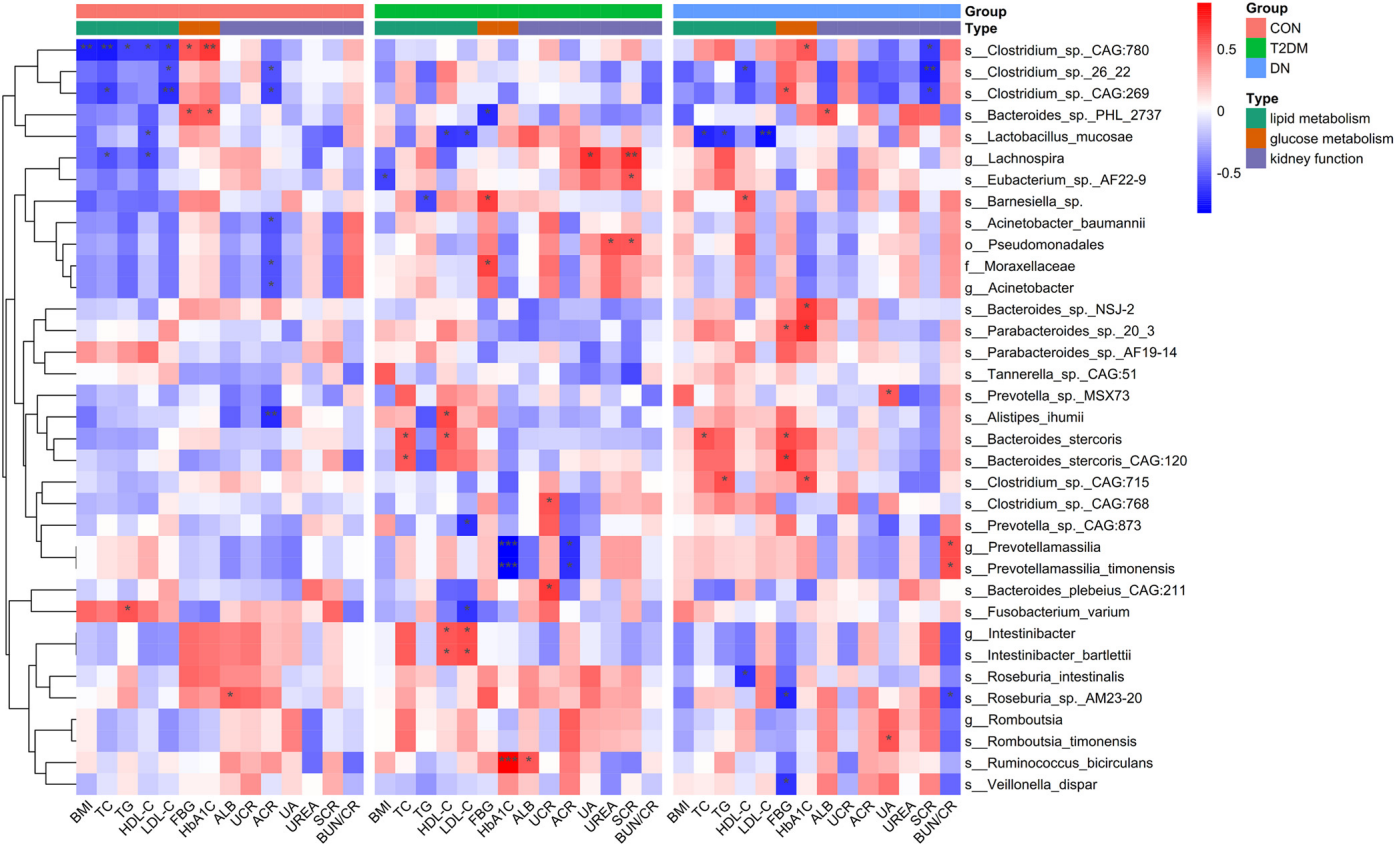
Spearman correlation analysis regarding the association between the differential species and clinical indexes. Red and blue indicate positive and negative correlations, respectively. *, *P* < 0.1; **, *P* < 0.05; ***, *P* < 0.005).

The correlations between differential species and diverse clinical indexes were estimated by the Spearman correlation analysis (Fig. 4). The clinical indexes were mainly divided into three categories: lipid metabolism (BMI, TC, TG, HDL-C, and LDL-C), glucose metabolism (FBG and HbA1C), and kidney function (ALB, UCR, ACR, UA, urea, SCR, and BUN/CR). In the CON group, *Clostridium* sp. CAG_780 was negatively correlated with BMI (*P* < 0.05) and TC (*P* < 0.05) and positively associated with HbA1C (*P* < 0.05). *Clostridium* sp. CAG_269 was negatively associated with LDL-C (*P* < 0.05). Regarding T2DM patients, *Lachnospira* presented a positive correlation with SCR (*P* < 0.05). *Prevotellamassilia* and *P. timonensis* were correlated with HbA1C (*P* < 0.005), while *R. bicirculans* showed a positive association with HbA1C (*P* < 0.005). Negative association was observed between *Clostridium* sp. 26_22 and SCR (*P* < 0.05) in DN patients, similar to *L. mucosae* and LDL-C in DN patients (*P* < 0.05).

### Potential diagnostic biomarkers for DN associated with the gut microbiota.

**FIG 5:**
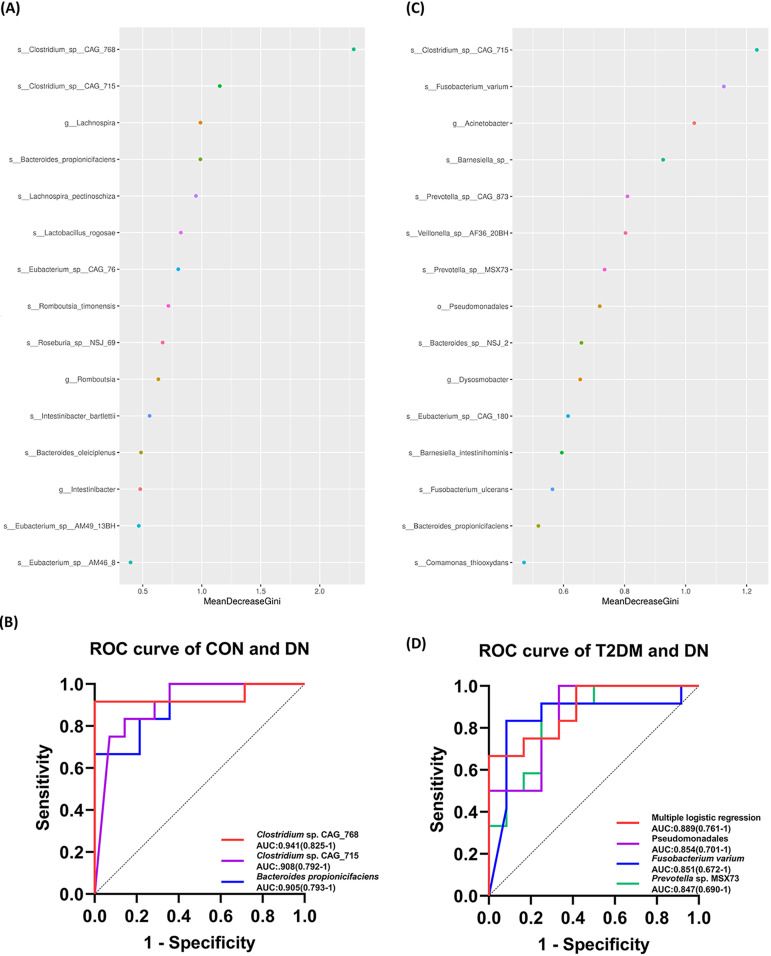
Gut microbiota biomarkers distinguishing the DN group from the CON and T2DM groups. (A) Random forest analysis of distinguishing species between the DN and CON groups, showing the 15 species with the highest contributions. (B) ROC curve differentiating the DN group from the CON group based on species with excellent effect. (C) Random forest analysis of distinguishing species between the DN and T2DM groups, showing the 15 species with the highest contributions. (D) ROC curve classifying the DN group from the T2DM group, based on species with excellent effect.

To further explore the application of the gut microbiota in the diagnosis of DN, the top 15 species distinguishing the DN group from the CON (Fig. 5A) and T2DM (Fig. 5C) groups were screened through mean decrease Gini (MDG)-based random forest analysis. Next, receiver operating characteristic (ROC) curve analyses were carried out according to the relative abundance of the top 15 species. Regarding to the CON and DN groups, the AUCs were 0.941, 0.905, and 0.908 for *Clostridium* sp. CAG_768, Bacteroides propionicifaciens, and *Clostridium* sp. CAG_715, respectively (Fig. 5B). We calculated the AUC of renal-function-related indexes in the present study. The diagnostic efficacy of the bacterial biomarkers was better than those of ACR, ALB, UCR, urea, SCR, and BUN/CR (Table S4). The AUC of the top 15 species was greater than 0.7 for association with T2DM and DN. Among them, *Pseudomonadales*, *F. varium*, and *Prevotella* sp. MSX73 were significantly associated with T2DM and DN, with AUCs of 0.854, 0.851, and 0.847, respectively. In order to further improve the differential effect, the ROC curve was presented by the logistics regression analysis of the above-mentioned species, with the highest AUC equal to 0.889 (Fig. 5D). The AUC of the bacterial biomarkers for T2DM and DN was higher than that of ACR, ALB, UCR, UA, urea, SCR, and BUN/CR (Table S4). Accordingly, the gut microbiota might be a well candidate for the diagnosis of DN. However, more cohort studies with larger sample sizes are warranted for validation.

### Function of the gut microbiota between the CON, T2DM, and DN groups.

**FIG 6:**
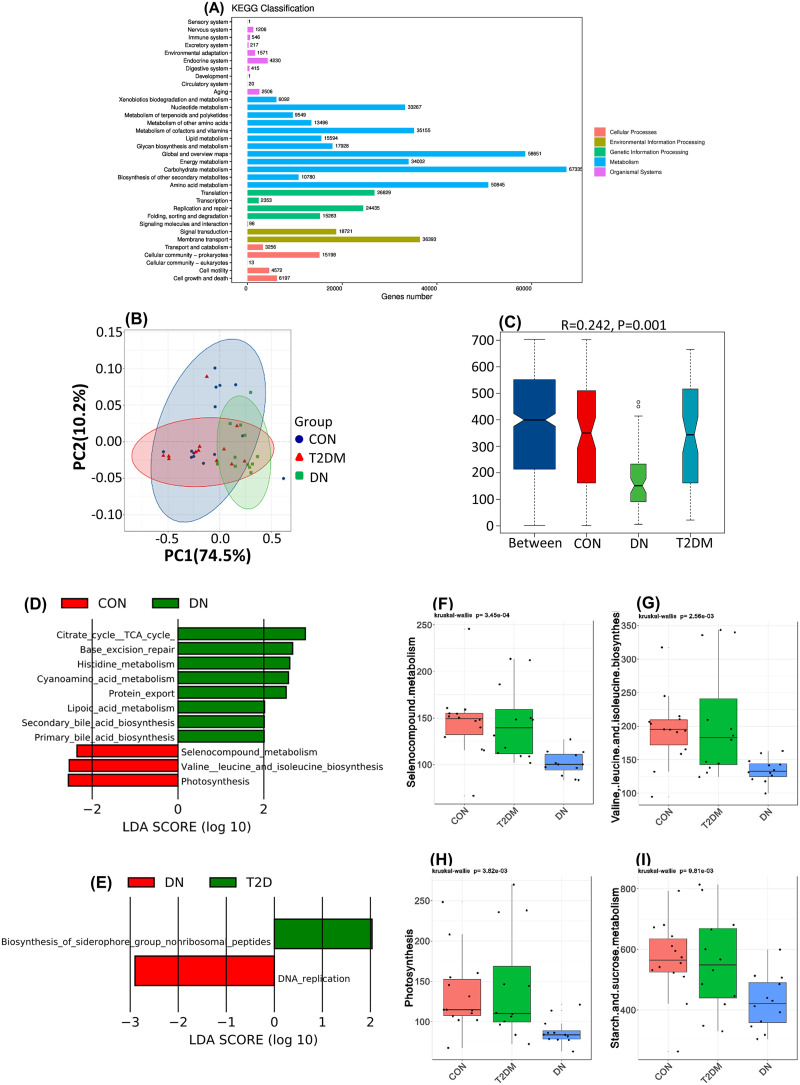
Function of the gut microbiota. (A) Gene numbers of KEGG classification in gut microbiota. (B) PCoA of gut microbiota function between the CON, T2DM, and DN groups. (C) ANOSIM analysis of gut microbiota function between the CON, T2DM, and DN groups. (D) LEfSe analysis of gut microbiota functions between the CON and DN groups. (E) LEfSe analysis of the gut microbiota functions between the T2DM and DN groups. (F to I) Gut microbiota function with a decreasing trend between the CON, T2DM, and DN groups.

To explore the role of the gut microbiota in DN and identify potential key genes or metabolites, we performed metabolic pathway analysis based on metagenomic sequencing (Fig. 6A). The results of the function clusters had shown that the gut microbiotas were mainly involved in biomolecular metabolisms, such as carbohydrates, amino acids, cofactors, vitamins, nucleotides, and lipids. They also played an important role in life activities, such as gene translation, transcriptional repair of proteins, signaling, and membrane transport. PCoA based on ANOSIM (Fig. 6C) using the Bray-Curtis dissimilarity matrix (Fig. 6B) suggested that the gut microbiota function was significantly altered in DN patients compared with CON and T2DM patients. A total of 126 specific pathways related to DN were estimated by Kruskal-Wallis test between the three groups. Compared with the CON group (Fig. 6D), the gut microbiota was enriched in lipoic acid metabolism, histidine metabolism, protein export, base excision repair, primary bile acid biosynthesis, secondary bile acid biosynthesis, cyanoamino acid metabolism, and tricarboxylic acid cycle functions in the DN group. Compared with that in patients with T2DM (Fig. 6E), DNA replication was significantly upregulated. Moreover, the biosynthesis of siderophore group nonribosomal peptides was significantly lower in patients with DN (Fig. 6E). In addition, decreased selenium compound metabolism was observed in T2DM and DN patients (Fig. 6F). Similar findings were obtained regarding branched-chain amino acid (BCAA) biosynthesis (Fig. 6G), photosynthesis (Fig. 6H), and starch and sucrose metabolism (Fig. 6I), which might be associated with DN progression.

## DISCUSSION

DN is a serious kidney disease and a major complication of diabetes. The pathogenesis of DN is complex and is usually influenced by multiple factors. The gut microbiota has been found to play an important role in DN. In this study, we utilized metagenomic sequencing to analyze the structure and function of the gut microbiota in T2DM and DN patients. Although no significant differences in gut microbiota diversity and richness were observed, the butyrate-producing bacteria and potential probiotics were demonstrated to be significantly decreased in the T2DM and DN groups. The significant correlation between the gut microbiota imbalance was closely correlated with the clinical indexes related to lipid metabolism, glucose metabolism, and renal function. Moreover, *Clostridium* sp. CAG_768, *B. propionicifaciens*, and *Clostridium* sp. CAG_715 could be used as gut microbiota biomarkers to distinguish CON from DN, while *Pseudomonadales*, *F. varium*, and *Prevotella* sp. MSX73 could be used to distinguish T2DM from DN. In addition, gut microbiota functions changed obviously and might influence the progression of DN. Nevertheless, the regulatory mechanisms of the gut microbiota in T2DM and DN remain largely unclear.

The correlation between gut microbiota α diversity and DN is currently controversial due to inconsistent findings from diverse studies. Du et al. have reported that the richness of the gut microbiota in DN patients was significantly lower than that in healthy controls, while the diversity of the gut microbiota did not change (23). A previous study has shown that the diversity of the gut microbiota in the DN group was not different from the control group, whereas the gut microbiota richness of the DN group was much higher than that of theT2DM group (17). However, a systematic review of 42 studies on the gut microbiota in patients with T2DM has suggested that there was no significant correlation between α diversity of the gut microbiota and T2DM (24). Similarly, we have demonstrated that there is no significant difference in α diversity of gut microbiota between the CON, T2DM, and DN groups. Moreover, the gut microbiota structure is consistent with the findings in a previously published study, which showed it to be mainly composed of *Bacteroidetes*, *Firmicutes*, *Proteobacteria*, and *Actinobacteria* (25). Moreover, the B/F ratio of gut microbiota is increased in patients with T2DM and DN. It has been documented that the B/F ratio of the gut microbiota in patients with T2DM is significantly increased (26, 27). The B/F ratio in the rodent model of DN also shows an elevated trend (28, 29). As a result, the decrease of *Firmicutes* may be a common phenomenon in the gut microbiota of patients with T2DM and DN. It has been shown that severe insulin resistance in T2DM patients was found to be associated with fewer *Prevotella* and more *Bacteroides* organisms (30). *Bacteroides* is a major contributor to kidney inflammation through LPS-Toll-like receptor 2/4 (TLR2/4) signaling pathway (31, 32). Similarly, we have found that the abundance of *Prevotella* was the highest in the CON group, while *Bacteroides* was the dominant genus in the T2DM and DN groups at the genus level, which is also consistent with the findings in the DN and CKD groups (33). Accordingly, the transformation of dominant bacteria may play an important role in promoting DN progression.

SCFAs can inhibit the proliferation of glomerular mesangial cells induced by high glucose and LPS. It reduces the production of reactive oxygen species (ROS), malondialdehyde (MDA), and inflammatory factors but increases the level of superoxide dismutase (SOD) and protects kidney function. Butyrate can also improve angiotensin II-mediated kidney injury by affecting the urinary protein, glomerular sclerosis, renal fibrosis, and inflammatory factor production (34). Reduced probiotics and butyrate-producing bacteria are common in T2DM patients (35). Similar findings have been obtained for prediabetic patients (36). Du et al. have also demonstrated that the SCFA-producing bacteria in the gut microbiota of DN4 patients were significantly lower than those in healthy controls, such as *Roseburia*, *Faecalibacterium*, *Blautia*, and *Subdoligranulum* (23). Tao et al. have reported that the SCFA-producing bacteria *Coprococcus* and *Lachnospira* in the gut microbiota of T2DM and DN patients were also significantly reduced (17). Similar to the case with previous reports, the LEfSe analysis in our study has shown that the intestinal beneficial bacteria *Lachnospira*, *Romboutsia*, and *Intestinibacter*, as well as strains of the butyrate-producing bacteria *Roseburia*, *Clostridium*, *Eubacterium*, and *Fusobacterium*, were significantly reduced in T2DM and DN patients. Taken together, the findings show that the decrease of SCFA-producing bacteria may promote the development of DN. As a high-yield butyrate-producing bacterium, *R. intestinalis* is significantly reduced in the gut microbiotas of patients with various diseases, including T2DM. It plays an important role in maintaining the stability of the intestinal environment by regulating inflammation and autoimmunity (37). Colonization of *R. intestinalis* in the gut can reduce inflammation and alleviate atherosclerosis by producing butyrate in mouse models (38) and can enhance the intestinal barrier to improve alcoholic fatty liver (39). In the present study, *R. intestinalis* was decreased in the gut microbiotas of patients with T2DM and DN, suggesting a crucial role of *R. intestinalis* in T2DM and DN. Nonetheless, more future studies are encouraged to estimate the role of those high-yield butyrate-producing bacteria in T2DM and DN, particularly *R. intestinalis*.

In addition, several strains enriched in the T2DM or DN group have attracted our attention. The enrichment of *Lactobacillus* has been proved to be related to metformin administration in T2DM patients (40). We have also found obvious enrichment of *L. mucosae* in the gut microbiota of T2DM patients. Although most of the included T2DM patients have been administered metformin, whether the increased *L. mucosae* is associated with metformin administration in T2DM is unclear due to relatively small sample size in this study. *V. dispar* is also increased in T2DM patients, and this organism can convert lactic acid to propionic acid (41). It has been documented that the higher the concentration of propionic acid in feces is, the higher the risk of T2DM occurring is (42). Nonetheless, the abundance of *V. dispar* in prediabetic patients has been found to be decreased in a previous study (43). In our study, the modifying effect of *V. dispar* on glucose metabolism needs to be extensively explored. The abundance of two strains of *B. stercoris* was found to be elevated in patients with DN. Most interestingly, the abundance of *B. stercoris* is also remarkably increased in the gut microbiota of children with type 1 diabetes (44). It has been shown that *B. stercoris* isolated from the feces of T2DM patients combined with a high-fat diet can significantly increase body weight, blood glucose, and inflammatory cytokines (tumor necrosis factor alpha [TNF-α], interleukin 6 [IL-6], and MCP-1) in mice, whereas the precise mechanism is not clear yet (45). The mechanism of *B. stercoris* in regulating DN needs more in-depth research.

Accumulated data have supported the notion that the gut microbiota is closely related to metabolic indexes, such as HbA1c, SCR, UA, ALB, and BUN. Karlsson et al. have clarified that the elevated *Lactobacillus* level in the gut microbiota of T2DM patients was positively correlated with HbA1c and FBG, while the decreased *Clostridium* level was negatively associated with HbA1c and FBG (46). However, our study has demonstrated that *Clostridium* sp. CAG_780 was positively associated with HbA1c. Tao et al. have reported a negative relationship of butyrate-producing bacteria (*Faecalibacterium*, *Lachnoclostridium*, and *Roseburia*) with HbA1c (17). In this study, we have found that the butyrate-producing bacteria enriched in the CON group show weak associations with HbA1c and FBG. A previous study has demonstrated that *Parabacteroides* was negatively correlated with SCR and *Bacteroides* was negatively related to ACR (17). A study by Ren Z et al. has elaborated that SCR and BUN were positively correlated with butyrate-producing bacteria of *Blautia*, *Butyricimonas* and probiotics of *Akkermansia*, but negatively correlated with *Veillonella* and *Lactobacillus* in CKD (47). ALB is positively associated with *Barnesiella* but negatively related to *Veillonella* (47). In the present study, *Lachnospira* showed a positive correlation with SCR, while *Clostridium* sp. 26_22 was negatively associated with SCR. Accordingly, the effect of specific members of the gut microbiota on renal function indexes is not consistent, which needs to be further investigated in future studies.

Currently available data have suggested that gut microorganisms can be used as diagnostic biomarkers for DN. Du et al. have found 25 bacteria through LEfSe and Metastat analysis in DN patients, some of which might be useful diagnostic markers (23). Tao et al. have reported that DN and T2DM can be effectively distinguished by Escherichia-*Shigella* and *Prevotella*_9, with an AUC equal to 0.86 (17). Different types of kidney diseases can also be distinguished by the gut microbiota (48, 49). In the present study, the AUCs under the ROC curve of *Clostridium* sp. CAG_768, *B. propionicifaciens*, and *Clostridium* sp. CAG_715 were 0.941, 0.905, and 0.908, which predicted the important role of the gut microbiota in diagnosing DN. Apart from that, the combination of *Pseudomonadales*, *F. varium*, and *Prevotella* sp. MSX73 could help to distinguish T2DM from DN (AUC = 0.889). However, the diagnostic role of specific gut microbiotas is heterogenous among individuals. Large-scale multicenter, cross-regional, and cross-ethnic studies are necessary to estimate the role of the gut microbiota in predicting the diagnosis of DN.

Apart from the gut microbiota composition, the functions of the gut microbiota obviously vary in patients with T2DM and DN. Lipoic acid can reduce the level of urinary albumin and oxidative stress in patients with DN, improve antioxidant capacity, and protect kidney function (50). We have found elevated lipoic acid in DN patients, which suggests that the gut microbiota may affect drug metabolism as demonstrated in a previous study (51).

Enhanced histidine metabolism was observed in the DN group when evaluating the function of the gut microbiota. Increased imidazole propionate from histidine metabolism can lead to insulin receptor substrate degradation through the mTORC1 pathway, which causes insulin resistance by blocking the insulin signal transduction (52). It has been well demonstrated that imidazole propionate is closely associated with systemic inflammation (53). The active histidine metabolism in DN patients may be attributed to the production of imidazole propionic acid, thus promoting the progression of DN. The bile acid receptor farnesol X receptor (FXR) plays an important role in regulating kidney lipid metabolism, inflammation, fibrosis (54), and the pathogenesis of DN (21). We have observed enrichment of primary and secondary bile acid in DN patients, which suggests that the gut microbiota may regulate the pathophysiology of DN through bile acid metabolism depending on the FXR/TGR5 signaling pathway. The base excision repair is the main way to repair ROS-induced DNA damage (55). The enhancement of base excision repair of the gut microbiota in patients with DN may be a protective mechanism to reduce the cumulative damage caused by DNA damage. Hyperglycemia usually produces excessive ROS leading to DN (11). Selenium deficiency may lead to increased vascular complications and microalbuminuria in diabetic patients by increasing oxidative stress (56), while selenium supplementation can improve the clinical indicators of DN patients (57). Therefore, the reduction of selenocompound metabolism in DN patients may play a protective role in reducing the metabolism of selenium compounds and promoting the absorption and utilization of selenium. It has been well documented that increased intake of branched-chain amino acids (BCAA) in the diet will increase the risk of T2DM and insulin resistance (58). In this study, DN patients showed a weakening of BCAA synthesis, which may be attributed to the decrease of *Prevotella* essential for BCAA synthesis (59). The decreased BCAA synthesis in DN patients can reduce the risk of BCAA-induced insulin resistance and the development of DN. In addition, PCoA showed that there was a significant difference regarding the functions of the gut microbiota in T2DM and DN patients.

There are some drawbacks in this study. First, the conclusions should be interpreted with caution due to the relatively small sample size. Second, more clinical indexes are needed when estimating the role of the gut microbiota in T2DM and DN. Lastly, it is well known that drugs can affect the gut microbiota. Most participants in this study have taken hypoglycemic or antihypertensive drugs, which may lead to bacterial variations.

### Conclusion.

In summary, this study has provided some insight on the compositions and functions of the gut microbiota in T2DM and DN patients through metagenomic sequencing analysis. In particular, the gut microbiota plays a critical role in the pathogenesis of DN. Butyrate-producing bacteria (Roseburia intestinalis, *Clostridium*, and *Eubacterium*) and potential probiotics (*Lachnospira* and *Intestinibacter*) are significantly decreased in the gut microbiotas of T2DM and DN patients. Some members of the gut microbiota are closely associated with clinical indexes, such as BMI, TC, HbA1C, LDL-C, and SCR. In addition, some gut microorganisms can be used as predictors for the diagnosis of DN, including *Clostridium* sp. CAG_768, *B. propionicifaciens*, and *Clostridium* sp. CAG_715. Moreover, gut microbiota functions in DN mainly by regulating the citrate cycle, base excision repair, histidine metabolism, lipoic acid metabolism, bile acid biosynthesis, selenium metabolism, and branched-chain amino acid biosynthesis. Nevertheless, more studies are warranted to clarify the precise mechanisms of the gut microbiota in substance metabolism. Future studies are encouraged to identify novel diagnostic or therapeutic strategies for T2DM and DN by targeting the gut microbiome.

## MATERIALS AND METHODS

### Experimental design.

This was a case-control study, including 15 patients with type 2 diabetes mellitus (T2DM) without any complications and 15 patients with diabetic nephropathy (DN) admitted to the Department of Endocrinology of the First Affiliated Hospital, Weifang Medical University, from August 2020 to December 2020. Fifteen age-, gender-, and BMI-matched healthy controls (CON) were also enrolled. T2DM patients were diagnosed based on the diagnostic criteria proposed by the World Health Organization (WHO) in 1999 (60). DN was diagnosed according to the diagnostic criteria proposed by the Microvascular Complications Group of the Chinese Diabetes Society: diabetic patients with macroalbuminuria or retinopathy plus chronic kidney disease in any stages. Exclusion criteria were as follows: patients with special types of diabetes or gestational diabetes; tumor; liver, kidney, and other organs with severe damage; hyperhidrosis and other endocrine diseases; administration of antibiotics or probiotics in the past 1 month; diarrhea or other gastrointestinal diseases in the past 1 month; and history of gastrointestinal surgery. Informed consent was obtained from all participants. The study was approved by the Medical Ethics Committee of the First Affiliated Hospital, Weifang Medical University (approval no. 2016-273). All procedures were performed in accordance with the principles of the Declaration of Helsinki.

### Sample collection.

Fresh feces were collected from patients within 48 h of admission and from healthy controls in the same hospital. Urine was emptied before fecal collection to avoid urine contamination. The central part of feces (10 ± 5) g was collected and placed in a sterile collection tube. All samples were stored at –80°C within 2 h of collection.

### DNA extraction and library construction.

Total DNA extraction from feces of all samples was performed using an Agencourt AMPure XP (Beckman Coulter, Brea, CA) kit according to the manufacturer’s instructions. The concentration and purity of extracted DNA were determined by use of a NanoDrop 2000 UV-visible (UV-Vis) spectrophotometer (Thermo Scientific, Wilmington, DE). The quality of DNA was checked by 1% agarose gel electrophoresis. Extracted DNA (1 μg in a total volume of 52.5 μL) was added to a Covaris tube and then fragmented using a DNA shearing instrument (Gene, Shanghai, China). The fragmented DNA was used to prepare a DNA library according to the protocol of TruSeq Nano DNA LT sample preparation kit (Illumina, San Diego, CA). Then, the bridge PCR was performed. The PCR amplification mixture consisted of 25 μL of purified ligation DNA, 20 μL of enhanced PCR mix, and 5 μL of PCR primer cocktail. PCR amplification conditions were as follows: 95°C for 3 min, 8 cycles of 98°C for 20 s, 60°C for 15 s, and 72°C for 30 s, 72°C for 5 min, and a 4°C hold.

### Metagenomic sequencing.

PCR products were purified and sequenced on an Illumina sequencing platform (Illumina, San Diego, CA) according to the standard protocol of OBiO Technology Corp., Ltd. (Shanghai, China).

### Sequencing data processing.

The raw data were in the FASTQ format. Adapters were first excised using Trimmomatic1 (v0.36). Low-quality bases were filtered out to remove reads containing N bases (ambiguous bases). The postfiltered pair-end reads were aligned to the host genome using bowtie2 (v2.2.9), which was subsequently discarded. After obtaining valid reads, metagenome assembly was performed using MEGAHIT (v1.1.2). We used gaps inside the scaffold as breakpoints to interrupt the scaffold into new contigs (ScafContig). These new ScafContig with lengths of ≥200 bp (or 500 bp) were retained. Open reading frame (ORF) prediction of the assembled scaffolds was performed and translated into amino acid sequences by use of prodigal (v2.6.3). CDHIT (v4.5.7) 5 was used to construct nonredundant gene sets for genes predicted in all samples with clustering parameters set at values of 95% identity and 90% coverage. The longest gene in each cluster set was selected as the representative sequence for that gene set. Clean reads of each sample were aligned against the nonredundant gene set (95% identity) using bowtie2 (v2.2.9). The abundant information of the gene in the corresponding sample was counted. DIAMOND6 (v0.9.7) software was applied to align and annotate the representative sequences (amino acid sequences) of the gene set with the NR library, KEGG, COG, Swiss-Prot, and GO, respectively. The BLAST alignment parameter was set with an expected E value of 1e−5. The species annotation was also obtained through the taxonomic information database according to the NR library. The abundance of the species was calculated using the corresponding abundance of the genes. In order to illustrate the abundance profile at the corresponding taxonomy level, abundance statistics were determined at levels of domain, kingdom, phylum, class, order, family, genus, and species. The gene sets were compared with the CAZy database using the tool hmmscan (v3.1b2) to obtain the information for genes related to carbohydrate active enzyme. Additionally, the carbohydrate activity was calculated based on the sum of these gene abundances.

### Statistical analysis.

R software (v3.2.0) and GraphPad Prism software (v8.3.0) were adopted for data statistics. Principal-coordinate analysis (PCoA) and plotting of the abundance spectrum of species or the functional abundance spectrum were carried out using R software (v3.2.0). The equidistant matrix of PCoA was calculated and analyzed. PCoA statistical analysis was performed by use of analysis of similarity (ANOSIM). The linear discriminant analysis effect size (LEfSe) was used to mine species and functions with statistical differences. Spearman correlation analysis was performed to estimate the correlation between differential species and clinical indexes. For bacterial biomarker identification, a two-step method was adopted. First, the random forest R package was applied to select the top 15 bacterial biomarkers according to the mean decrease Gini (MDG) value based on all of the samples. Second, the receiver operating characteristic (ROC) curve of each bacterial biomarker was analyzed to evaluate the diagnostic value of gut microbiota for diseases by use of GraphPad Prism software. The area under the curve (AUC) was also calculated. Logistic regression analysis was carried out to construct ROC curves for multiple bacterial biomarkers. Data were presented as *--x *±* s* for continuous variables. One-way analysis of variance (ANOVA) or Kruskal-Wallis test was performed to evaluate data of normal distribution. For the comparison between pairwise data, the least significant difference (LSD) multiple-comparison *post hoc* test was applied in the one-way ANOVA, while Dunn’s multiple-comparison *post hoc* test was performed with the Kruskal-Wallis test. Enumeration data were expressed as percentages, which were analyzed by *χ*^2^ test. A *P *value of <0.05 was considered statistically significant.

### Data availability.

All sequencing data were deposited in the Genome Sequence Archive (GSA) database under accession number CRA007013.
