# Supplementary material for: Alterations of the Gut Microbiota in Patients with Diabetic Nephropathy
Source: Microbiol Spectr. 2022 Jul 14;10(4):e00324-22. doi: 10.1128/spectrum.00324-22 (PMC9430528; doi:10.1128/spectrum.00324-22)
Supplement: Supplemental file 1 — Supplemental material. Download spectrum.00324-22-s0001.pdf, PDF file, 0.2 MB [file spectrum.00324-22-s0001.pdf]

Supplementary tables

Table S1 The relative abundance of the top 15 bacteria of the gut microbiota in the CON, T2DM, and DN groups at the phylum level (%, $x \pm s$)

phylum	CON	T2DM	DN	*P* 值
Bacteroidetes	61.94±22.73	60.30±26.01	67.64±14.37	0.881
Firmicutes	28.98±16.40	27.42±18.80	24.15±9.97	0.729
Proteobacteria	5.11±7.94	8.79±9.49	4.27±5.29	0.525
Actinobacteria	0.34±0.26	1.47±2.30	1.07±1.18	0.443
Candidatus_Melainabacteria	0.55±1.03	0.32±0.99	0.06±0.08	0.245
Uroviricota	0.53±1.29	0.04±0.04	0.16±0.29	0.564
Fusobacteria	0.35±1.24	0.16±0.51	0.01±0.01	0.198
Tenericutes	0.24±0.63	0.02±0.06	0.15±0.19	0.167
Verrucomicrobia	0.10±0.17	0.05±0.04	0.18±0.32	0.889
Euryarchaeota	0.02±0.02	0.02±0.04	0.16±0.26	0.054
Lentisphaerae	0.00±0.00	0.00±0.00	0.10±0.35	0.196
Chlamydiae	0.05±0.13	0.01±0.01	0.01±0.01	0.295
Synergistetes	0.00±0.01	0.05±0.17	0.00±0.01	0.090
Spirochaetes	0.01±0.01	0.01±0.01	0.01±0.02	0.590
Crenarchaeota	0.02±0.07	0.00±0.00	0.00±0.00	0.111

Table S2 The relative abundance of the top 15 bacteria of the gut microbiota in the CON, T2DM, and DN groups at the genus level (%, x±s)

genus	CON	T2DM	DN	*P* 值
Bacteroides	17.94±14.18	26.67±17.41	29.22±15.23	0.109
Prevotella	27.31±30.01	11.35±20.20	16.28±21.11	0.429
Phocaeicola	10.13±8.80	12.80±9.26	9.59±7.42	0.538
Faecalibacterium	6.50±5.02	7.81±6.89	3.78±2.51	0.169
Alistipes	1.91±1.88	2.71±2.90	4.74±5.01	0.339
Parabacteroides	1.88±1.15	3.94±3.56	3.28±1.90	0.066
Clostridium	2.82±2.27	2.09±1.84	2.48±1.68	0.679
Roseburia	2.39±2.00	2.30±2.72	1.62±0.86	0.585
Eubacterium	1.98±1.37	2.02±2.67	1.66±2.31	0.417
Escherichia	2.07±6.21	2.63±4.62	0.61±0.66	0.052
Ruminococcus	1.60±0.89	1.10±0.99	1.63±1.08	0.156
Klebsiella	0.46±0.68	2.87±5.67	0.60±1.00	0.199
Blautia	1.03±0.79	1.12±1.06	1.00±0.75	0.942
Lachnospira	0.90±0.61	0.95±1.02	0.68±1.67*	0.022
Bifidobacterium	0.20±0.19	1.03±2.12	0.77±0.99	0.606

*P<0.05, compared with CON groups

Table S3 The relative abundance of the top 15 bacteria of the gut microbiota in the CON, T2DM, and DN groups at the species level (%, $\bar{x}$±s)

species	CON	T2DM	DN	*P* 值
Prevotella copri	22.13±25.77	8.24±16.86	11.08±18.47	0.197
Phocaeicola vulgatus	3.76±3.47	6.30±4.25	4.88±3.59	0.230
Faecalibacterium prausnitzii	4.31±3.38	5.12±4.31	2.56±1.66	0.177
Phocaeicola plebeius	4.40±6.30	3.06±5.33	1.89±4.81	0.374
Bacteroides uniformis	1.91±2.19	2.88±2.92	4.19±2.40	0.064
Bacteroides ovatus	1.89±2.31	2.98±3.02	2.73±2.32	0.379
Bacteroides fragilis	2.47±6.46	3.25±5.56*	1.24±0.66	0.034
Bacteroides stercoris	0.88±1.14	1.52±2.04	3.31±3.74*	0.031
Escherichia coli	2.06±6.17	2.62±4.59	0.61±0.66	0.051
Phocaeicola dorei	1.20±0.87	2.22±1.36	1.68±1.19	0.161
Bacteroides xylanisolvens	1.07±1.16	2.03±2.64	1.34±1.02	0.552
Bacteroides thetaiotaomicron	1.21±1.34	1.40±1.18	1.58±1.06	0.292
Bacteroides caccae	0.69±0.59	1.31±1.34	1.29±0.92	0.115
Parabacteroides merdae	0.64±0.44	1.33±1.48	0.98±0.65	0.316
Parabacteroides distasonis	0.52±0.35	1.05±0.80	1.06±0.71	0.606
[Eubacterium]_rectale	0.01±0.01	0.01±0.01	0.01±0.01	0.239
Lachnospiraceae_bacterium	0.01±0.01	0.01±0.00	0.01±0.01	0.386
Prevotella_sp.	0.01±0.01	0.00±0.01	0.01±0.01	0.184
Bacteroides_sp.	0.00±0.00	0.01±0.01	0.01±0.01	0.750
Eubacterium_sp.	0.01±0.01	0.01±0.01	0.01±0.01	0.266
Phocaeicola_coprocola	0.00±0.00	0.01±0.01	0.01±0.01	0.975
Clostridia_bacterium	0.01±0.01	0.00±0.00	0.01±0.01	0.309

Klebsiella_pneumoniae	0.00±0.00	0.01±0.03	0.00±0.01	0.166
Ruminococcaceae_bacterium	0.01±0.01	0.00±0.00	0.01±0.00	0.154
Lachnospira_eligens	0.01±0.01	0.01±0.01	0.00±0.01	0.090
Alistipes_putredinis	0.00±0.00	0.00±0.01	0.01±0.02	0.149
Faecalibacterium_sp.	0.01±0.00	0.01±0.01	0.00±0.00	0.124
Roseburia_inulinivorans	0.01±0.01	0.00±0.01	0.00±0.00	0.167
Bacteroides_eggerthii	0.00±0.00	0.00±0.00	0.01±0.01*	0.042
Bacteroides_cellulosilyticus	0.00±0.00	0.00±0.00	0.01±0.02	0.220

*P<0.05, compared with CON groups

Table S4 AUC and 95%CI of renal function related indexes distinguishing DN from CON and T2DM

Parameters	CON-DN		T2DM-DN	
	AUC	95%CI	AUC	95%CI
ACR	0.881	0.718 - 1.000	0.826	0.647 - 1.000
ALB	0.815	0.622 - 1.000	0.785	0.592 - 0.977
UCR	0.839	0.642 - 1.000	0.757	0.556 - 0.957
UA	0.929	0.830 - 1.000	0.635	0.402 - 0.869
UREA	0.738	0.532 - 0.944	0.583	0.338 - 0.829
SCR	0.637	0.416 - 0.857	0.520	0.277 - 0.764
BUN/CR	0.518	0.285 - 0.750	0.524	0.282 - 0.766
